# American Society of Dental Surgeons

**Published:** 1841

**Authors:** 


### AMERICAN SOCIETY OF DENTAL SURGEONS.

The formation of this Society seems to have infused new spirit into the profession. Since its formation, we have received communications from many of its most intelligent and respectable members, residing in various and remote parts of the country, expressing their high gratification at the establishment of an institution so eminently calculated to advance the science of Dental Surgery, and a determination to become members of it. We are glad that it meets with such general approbation ; it augurs well for its perpetuity and future usefulness.

The favour which the Society has met with among the members of our own immediate profession, is little greater than what it has received from the medical.

We copy the following from " *The Boston Medical and Surgical Journal*," for October 21st, 1840, and one of the most ably conducted and popular medical periodicals of the day.

" American Society of Dental Surgeons.—At a meeting of Dentists, assembled in the city of New-York, on the 18th of August, a constitution was adopted, and all dentists of reputation will hereafter probably belong to the Society—the object being to elevate the profession, and to establish uniformity in practice upon scientific principles. Horace H. Hayden, M. D., of Baltimore, was elected President ; Dr. J. F. Flagg, of Boston, E. Parmly, of New-York, and E. B. Gardette, Vice Presidents. Dr. Parmly was appointed an agent to petitionthe Legislature of the State of New-York, asking for a charter, with power to confer the degree of *Doctor of Dental Surgery*. The next meeting will take place on the 2nd Tuesday of August, 1841, at the U. S. Hotel, Philadelphia. The above intelligence was gleaned from the last number of the American Journal of Dental Science, a periodical which we are always glad to receive. It is creditable to the Dentists of the United States, that they have succeeded in organizing themselves into a regular College—the good effects of which we trust will soon be manifest. If the literati, the philosophers and the cultivators of general science, in this country, had half the indomitable perseverance of the gentlemen who composed this convention, the many attempts made within the last two years to establish a National Association, would not have turned out to be mortifying abortions."

The editor of the same publication, for October 28th, after adverting to the appointment of members of the Society to prepare dissertations, together with the subjects thereof, that were respectively assigned to them, thus remarks :

" Physicians will be as glad to possess these discourses as the most enthusiastic dentist ; and we cannot feel otherwise than solicitous to have them extensively circulated as a distinct work, instead of being confined to the pages of the Dental Journal. Let every person who has defective teeth, have an opportunity of reading the opinions of the best operators in the land."

We are gratified to learn that the efforts of the Society to advance the science, are thus favourably regarded, and are happy in being able to inform such as may feel an interest in its doings, that it is the intention of it to publish its transactions, together with the essays and dissertations that may be read before it, in a volume by themselves.

